# Traumatic spinal cord injury in the north-east Tanzania – describing incidence, etiology and clinical outcomes retrospectively

**DOI:** 10.1080/16549716.2017.1355604

**Published:** 2017-08-31

**Authors:** Haleluya Moshi, Gunnevi Sundelin, Klas-Göran Sahlen, Ann Sörlin

**Affiliations:** ^a^ Department of Community Medicine and Rehabilitation, Physiotherapy, Umeå University, Umeå, Sweden; ^b^ Faculty of Rehabilitation Medicine, Physiotherapy Department, Kilimanjaro Christian Medical University College, Moshi, Tanzania; ^c^ Department of Public Health and Clinical Medicine, Epidemiology and Global Health, Umeå University, Umeå, Sweden; ^d^ Department of Nursing, Umeå University, Umeå, Sweden

**Keywords:** Traumatic spinal cord injury, etiology, rural, Tanzania, Africa

## Abstract

**Background**: Causes, magnitude and consequences of traumatic spinal cord injury depend largely on geography, infrastructure, socioeconomic and cultural activities of a given region. There is a scarcity of literature on profile of traumatic spinal cord injury to inform prevention and rehabilitation of this health condition in African rural settings, particularly Tanzania.

**Objective**: To describe the incidence, etiology and clinical outcomes of traumatic spinal cord injury and issues related to retrospective study in underdeveloped setting.

**Methods**: Records for patients with traumatic spinal cord injury for five consecutive years (2010–2014) were obtained retrospectively from the admission wards and health records archives of the Kilimanjaro Christian Medical Center. Sociodemographic, cause, complications and patients’ condition on discharge were recorded and analyzed descriptively.

**Results**: The admission books in the wards registered 288 new traumatic spinal cord injury cases from January 2010 to December 2014. Of the 288 cases registered in the books, 224 were males and 64 females with mean age 39.1(39.1 ± 16.3) years and the majority of individuals 196(68.1%) were aged between 16 and 45 years. A search of the hospital archives provided 213 full patient records in which the leading cause of injury was falls 104(48.8%) followed by road traffic accidents 73(34.3%). Cervical 81(39.9%) and lumbar 71(34.74%) spinal levels were the most affected. The annual incidence for the Kilimanjaro region (population 1,640,087) was estimated at more than 26 persons per million population. The most documented complications were pressure ulcers 42(19.7%), respiratory complications 32(15.0%) and multiple complications 28(13.1%). The mean length of hospital stay was 64.2 ± 54.3 days and the mortality rate was 24.4%.

**Conclusion**: Prevention of traumatic spinal cord injury in North-east Tanzania should consider falls (particularly from height) as the leading cause, targeting male teenagers and young adults. Pressure ulcers, respiratory complications, in-hospital mortality and availability of wheelchairs should be addressed.

## Background

Traumatic spinal cord injury (TSCI) is a sudden forceful damage to the spinal nerves resulting in temporary or permanent paralysis, bladder and bowel dysfunction and autonomic imbalance among other consequences [1,2]. A person with spinal cord injury is at immediate risk of respiratory and cardiac failure which may lead to death in the acute phase [3]. Those who survive the acute phase, faces a lifelong risk of secondary complications such as pressure ulcers, urinary tract infections, deep venous thrombosis, contractures, chronic pain and spasms [1,4].

Trauma to the spinal cord may result from a road traffic accident (RTA), fall, assault and recreational or occupational accident [1,5]. The World Health Organization informs that up to 90% of all spinal cord lesions are due to trauma and that the leading cause globally is RTA [1]. Countries and regions where RTA is the leading cause of TSCI are mostly of high income and in African cities and towns where there is dense traffic of four, three and two-wheeled motorized vehicles [1,5]. Worldwide, falls are the second leading cause of TSCI although in some parts of India, Nepal, South Asia, Estonia and Ireland it is reported the first leading cause [6].

In some regions, change in etiological pattern with time are reported. For example, although RTA is still the leading cause of TSCI in the USA a considerable increase in fall incidents have been reported in recent years [7]. At the same time assaults are the major cause of TSCI in South Africa [6,8]. Variation of leading causes of TSCI among countries and regions is explained by the fact that the predominating infrastructure, socioeconomic and cultural activities from which such trauma may result, are geographically not homogenous [5]. Except for the emerging motorbike use as public transport [9] rural Africa and Tanzania have fewer motorized vehicles and so causes of TSCI other than RTA can be anticipated.

Review of available reports from different countries indicates that between 170,000 and 250,000 of the world population suffer TSCI annually, with incidence rate of 23 persons per million population per year [5]. Country-specific incidence reports on TSCI are few, differ significantly and are mostly documented from high income countries [10]. Recently, New Zealand and the USA registered the highest annual incidence rates of 96 and 54 persons per million population, respectively [11]. A systematic review of the available incidence studies for low income countries ascertained that 25.5 persons per million population suffer TSCI annually [12]. Two prospective hospital-based studies conducted in Gaborone, Botswana and Cape Town, South Africa reported TSCI annual incidences of 13 and 75.6 persons per million population respectively [8,13]. Higher incidence rates can be anticipated in the unreported low income regions due to existence of unsafe occupational socioeconomic and cultural activities within a risky infrastructure [14]. There is a scarcity of reports from rural Africa regarding the magnitude, etiology and clinical outcomes of TSCI. Reporting the epidemiology and clinical outcomes of TSCI is crucial in order to inform health and rehabilitation personnel of the areas to target for a successful prevention and rehabilitation of persons with this condition [8].

The aim of this study is to describe the situation of TSCI within a low income and resource constrained Kilimanjaro rural region in Tanzania as well as addressing issues related to clinical outcomes in such settings. It is expected that this information will aid in planning and evaluating TSCI prevention, as well as alerting future researchers of issues related to retrospective studies in this and similar settings.

## Methods

### Study design

This is a hospital-based retrospective descriptive study, whereby demographic and clinical outcome information is collected from patient records for five consecutive years (2010–2014). Although this design suffers from a risk of losing some cases and necessary records, it is still worth undertaking in this setting as there are no previous publications on the studied subject [15].

### Data sources, collection and analysis

Names, age, sex and addresses of all patients who acquired TSCI for the 5 years (2010–2014) were collected from the admission books in the hospital wards. The list of the registered patient details (name, sex, age and address) were submitted to the health records department for retrieval of the each patient’s full clinical record. Of the 288 cases obtained from the admission books in the hospital wards, 75 were missing from the health records archives hence achieved sample was 213. The contents of the international SCI core data set [16] was incorporated in a constructed data collection sheet to capture data from the obtained 213 patients’ full records. This data sheet comprised of sociodemographic characteristics, cause of injury, spinal level, length of hospital stay (as per international SCI data set) with addition of complications and status on discharge. Analysis was performed descriptively using computer software (SPSS version 22) and results presented as numbers and percentages. Age was grouped by 15 increments and results for age and LOS are presented by mean, standard deviation, median and range. Computation of the significance of mean differences among categorical variables against LOS were performed by one-way analysis of variance (ANOVA) while association between two categorical variables is computed by cross tabulation and chi-squire with confidence interval (CI = 95%). Incidence is calculated for a specified region (Kilimanjaro) from which most persons were received 218 (75.7%) with a known population size [17]. In this study, we considered the whole population as at risk of TSCI regardless of age or sex due to the array of causes and calculation for incidence rate included the Kilimanjaro population for year 2012 which is the midpoint for 2010–2014. The incidence rate is calculated by taking the number of new cases from Kilimanjaro region in a given year over the 2012 population size.

## Results

The results for this study covered two areas. The first section is results based on all persons with TSCI in the north-east Tanzania (Kilimanjaro, Arusha and other surrounding regions) admitted to KCMC (Hospital) over 5 years (n = 288). The second section is epidemiological analysis involving persons specifically from the Kilimanjaro region (n = 218). Due to the inconsistency of hospital recording and monitoring of neurological status of each patient from admission to discharge, data on completeness of the injury, neurological level and functional outcomes were insufficient for analysis. Complications, mortality and patients´ ambulatory state on discharge are used for clinical outcomes in this report. The reported incidence was calculated based on the 2012 population of Kilimanjaro (as the midpoint for 2010–2014) and by in cooperating the 75 missing case records as their sex, age and address were obtained from the ward admission books.

### Socio-demographic characteristics of persons with TSCI – North-East Tanzania

**Figure 1.**
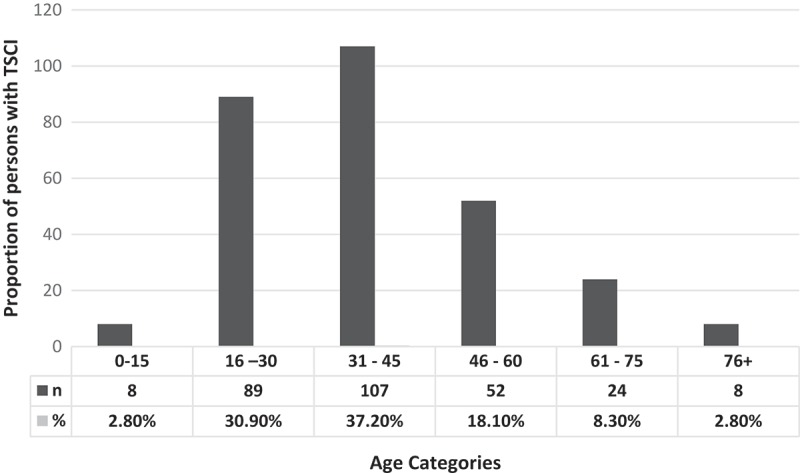
Distribution of persons with TSCI by age category.

This study included 288 persons registered TSCI from north-east Tanzania for the specified 5 years (2010–2014). Of the 288 cases, 75 case-files were missing from the health records archives and so they were only included in the calculation of incidence rate, proportion by region, sex and age. For the rest of the analysis the 75 cases were dropped out reducing the sample size to 213. Based on the 288 cases the majority of the TSCI were from Kilimanjaro 218(75.7%), Arusha 42(14.6%) and other regions 26(9.7%). The mean age was 39.1 ± 16.3 (range 5–92 years) and males were more affected than female with a male to female ratio of 3.5:1. Of the 213 cases whose full reports were obtained from health records archives 196(68%) aged between 16 and 45 years and the majority 199(93.4%) had plain x-rays as radiological investigation while few CT scans were conducted 14(6.6%). These investigations revealed that most injuries occurred at the cervical 81(28.1%) and lumbar 71(24.7%) regions. Fewer incidents were registered at thoracic 46(16.0%) and fewest 15(5.2%) had spinal cord injury without obvious radiological abnormality (SCIWORA). Distribution of the involved persons by age is summarized in Figure 1.

### Etiology of TSCI – North-East Tanzania

**Figure 2.**
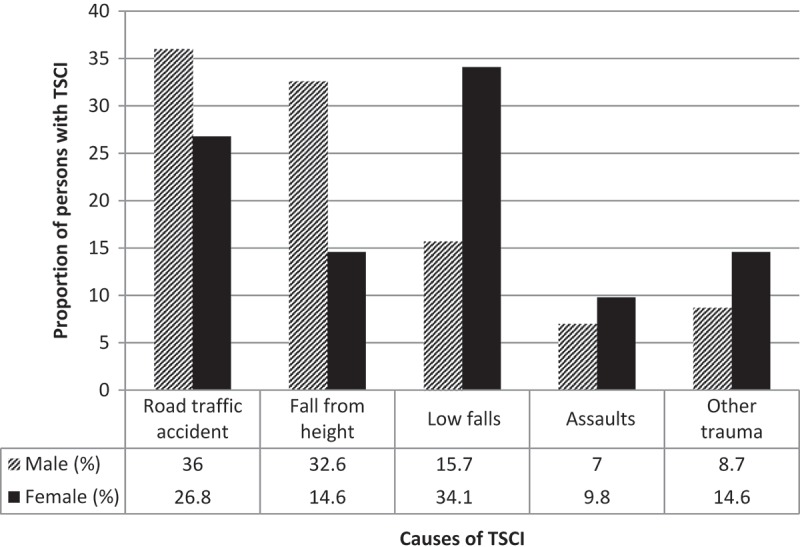
Causes of TSCI by sex (n = 213).

The leading cause of TSCI in this study were falls 104(48.8%) followed by road traffic accidents 73(34.3%). Falls included fall from height (more than 1 meter above the surface) and other falls (while walking, running or with a load on the head). When falls were analyzed further, fall from height was more frequently reported among male 56(32.6%) as compared to female 6(14.6%), whereas other falls (low falls) were more reported among female 14(34.1%) as opposed to male 27(15.7%) and this etiological difference with sex was found to be significant (p = 0.019). Other traumatic causes were being hit by animals (especially cattle) or a heavy load landing on a person 20 (9.4%) and assaults 16 (7.4%) as presented in Figure 2.

### In-hospital complications, length of stay, mortality and status on discharge

Pressure ulcers were the most frequently documented 42(19.7%), followed by respiratory complications 32(15.0%), multiple complications 28(13.1%) and urinary tract infections 17(8.0%). Fewest incidents of deep venous thrombosis 9(4.2%) were registered.

The mean length of hospital stay (64.15 ± 54.21 days) was significantly higher for thoracic (88.5 ± 64.7 days) and lumbar (63.9 ± 53.7 days) as compared to cervical (53.4 ± 44.3 days) TSCI (p = 0.003). A significantly shorter hospital stay was registered for patients who had associated injuries (56.0 ± 52.8 days) as compared to those who did not (79.4 ± 53.8 days)and also shorter in the presence of complications (46.7 ± 37.8 days) as compared to those who did not have complications (75.7 ± 60.2 days). Patients who were wheelchair dependent spent longer time in the hospital (118.9 ± 54.1 days) as compared to those who died (59.4 ± 54.3), walked aided (51.4 ± 23.4 days), bed ridden (38.1 ± 45.5) and walked unaided (24.9 ± 18.9 days).

LOS was significantly shorter for persons who had associated injuries (56.0 ± 52.8) as compared to those who had TSCI only (79.4 ± 53.8). Almost one fourth 48 (24.4%) of all persons with TSCI died while in hospital. The majority of those who died being males 39(81.3%), and 29(69.5%) aged between 16 and 45 years. Furthermore, the majority of those who died had a cervical injury 22(27.2%) and pressure ulcers 16(33.3%) and no associated injuries 29 (69.1%). Table 1 summarizes the death incidents by sex, cause of injury, age group and level of spinal injury.

**Table 1.** Distribution mortality cases by sex, age group and level of TSCI.

	Alive (%) *n* = 165	Dead (%) *n* = 48	Total (%) *n* = 213	*p*-value
**Sex**				
Male	133 (80.6)	39 (81.2)	172 (80.8)	0.55
Female	32 (19.4)	9 (18.8)	41 (19.2)	
**Causes of TSCI**				
RTA	59 (35.8)	14 (29.2)	73 (34.3)	0.53
Fall from height	49 (29.6)	13 (27)	62 (29.1)	
Other falls	27 (16.4)	14 (29.2)	41 (19.2)	
Assaults	14 (8.5)	2 (4.2)	16 (7.5)	
Other causes	16 (9.7)	5 (10.4)	21 (9.9)	
**Age group**				
0 – 15	7 (4.2)	0 (0.0)	7 (3.3)	0.03
16 – 30	58 (35.2)	13 (27.1)	71 (33.3)	
31 – 45	61 (37.0)	16 (33.3)	77 (36.2)	
45 – 60	27 (16.4)	8 (16.7)	35 (16.4)	
61 – 75	7 (4.2)	8 (16.7)	15 (7.0)	
76+	5 (3.0)	3 (6.2)	8 (3.8)	
**Level of SCI**				
Cervical	59 (35.8)	22 (45.8)	81 (38.0)	0.33
Thoracic	38 (23.0)	8 (16.7)	46 (21.6)	
Lumbar	58 (35.2)	13 (27.1)	71 (33.3)	
*SCIWORA	10 (6.0)	5 (10.4)	15 (7.1)	

*SCIWORA is spinal cord injury without obvious radiological abnormality. In this case the individual has signs and symptoms of SCI but no derangement or injury seen in the radiological imaging.

**Figure 3.**
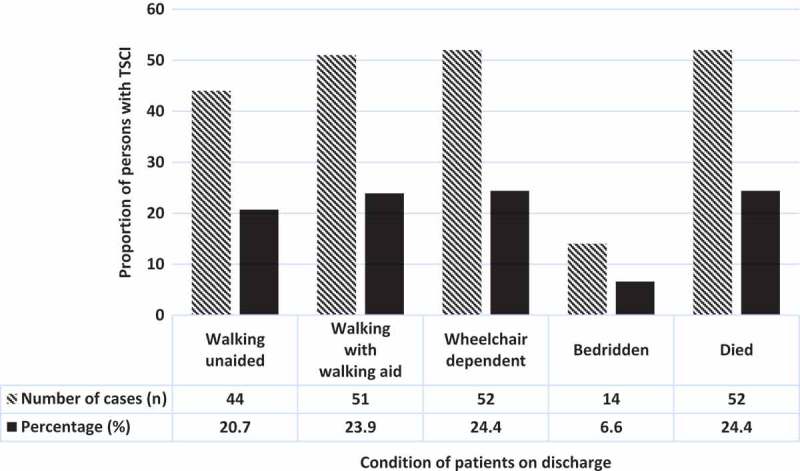
Condition of patients at discharge from the hospital (n = 213).

The majority of the patients became wheelchair ridden or needed a walking aid on discharge from the hospital. Figure 3 presents mortality and ambulation status of patients at discharge from the hospital.

**Figure 4.**
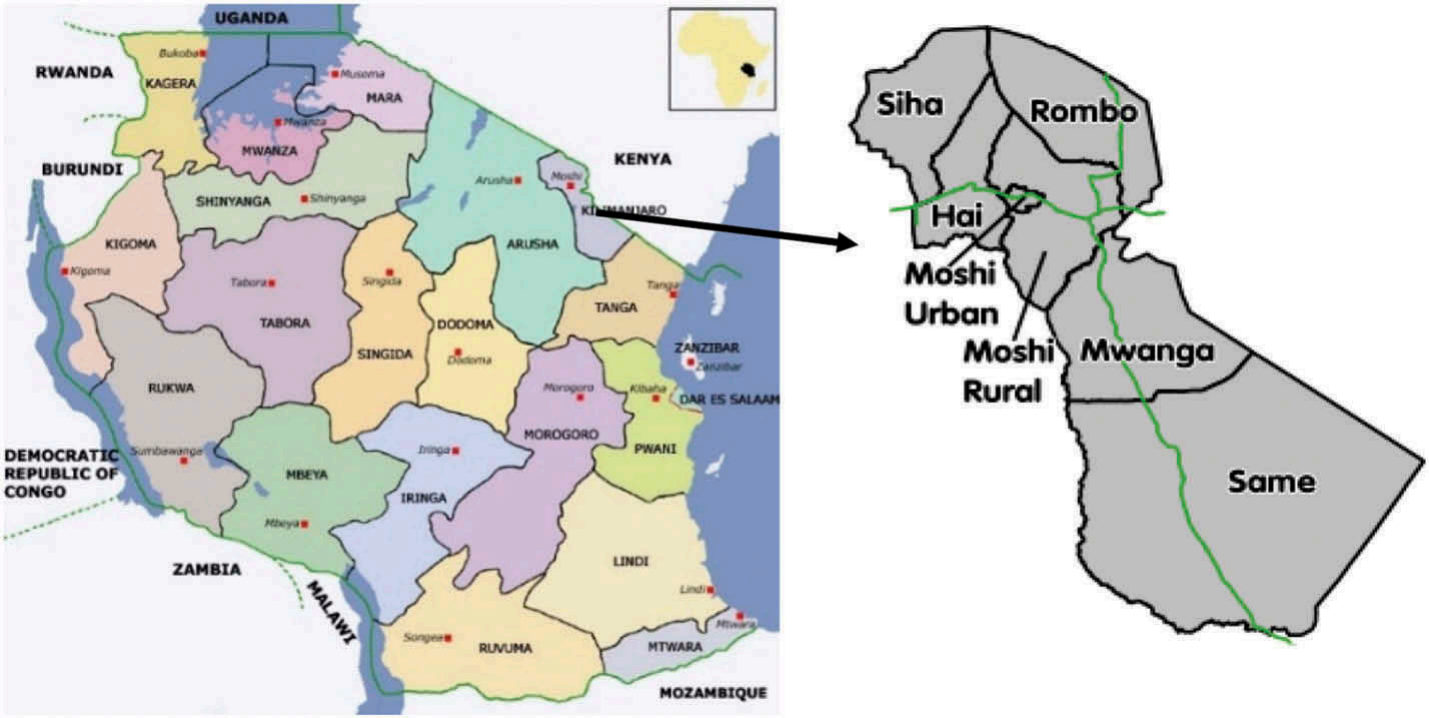
Tanzania (on the left) and Kilimanjaro region (on the right). **Source**: http://www.tpsftz.org/zoom.php?region=13&txt=Kilimanjaro and http://geography.about.com/library/cia/blctanzania.ht

### Estimated incidence rate of TSCI (in Kilimanjaro region)

The number of persons with TSCI (from Kilimanjaro region only) as registered in the ward admission books were 218. As shown in the Table 2, the annual incidence for Kilimanjaro region is estimated at 26 persons per million population and differs among sex, districts and age groups.

**Table 2.** Sex and age-specific crude annual incidence of TSCI in the Kilimanjaro region (n = 218).

Sex	Population size	*n* (%)	Incidence rate for 2010 – 2014 (Per 1,000,000 per year)
Men	793,140	171 (78.4)	43
Women	846,947	47 (21.6)	11
Overall	1,640,087	218 (100)	26
**Districts of the Kilimanjaro region**			
Hai and Siha	326,846	77 (35.3)	47
Moshi Rural and Rombo	727,700	89 (40.8)	24
Moshi Municipal	184,292	24 (11)	26
Same and Mwanga	401,249	28 (12.9)	13
**Age group**			
0–15	660,221	5 (2.3)	1
16–30	402,778	61 (28)	30
31–45	258,745	84 (38.5)	64
46–60	177,287	40 (18.3)	45
61–75	94,048	20 (9.2)	42
76 +	47,011	8 (3.7)	34

The incidence rate calculation is based on population from Tanzania census (2012) for Kilimanjaro region only (excluding Arusha and other regions). Population is taken at 2012 as the midpoint between 2010 and 2014. Except for Moshi Municipal, the rest are two districts combined as they are close to each other and share similar geographical and socioeconomic characteristics. Kilimanjaro region has six mostly rural districts and a small town – Moshi Municipal council (Figure 4).

## Discussion

This is the first study on magnitude and clinical outcomes of TSCI in the north-east region of Tanzania. Falls (particularly from height) were the leading cause of TSCI followed by road traffic accidents. Male teenagers and young adults aged between 16 and 45 years were the most affected. In hospital mortality was 24.4% and the commonest comorbidities were pressure ulcers and respiratory complications.

The estimated annual incidence reported in this study (more than 26 persons per million population) is relatively high as compared to the reported globally and in low income countries (23 and 25.5 persons per million population) respectively [5,12,18]. However there are other country and regional–specific studies that have reported higher annual incidences such as in Cape Town – South Africa and the United States which reports 75 and 54 persons per million population respectively [8,19]. Despite this incidence being higher than the average global incidence, it is obvious that it is still under-estimated because this was a retrospective study whereby some cases might have been missed [19]. some cases might have been missed, so the incidence in this report could be a reflection of a problem with a bigger magnitude. Furthermore, stable (non-serious) cases of TSCI are often admitted in the regional and district-designated hospitals, hence missed in this counting. There are no standby paramedics or emergency services in Tanzania for basic life support before and immediately on arriving at the hospital [20,21] therefore there is a high chance that severely injured individuals with TSCI die before they get to the hospital or at the emergence department before being admitted. Pre hospital mortality could also be due to delay of the injured person to be admitted to the appropriate health facility as in Nigeria [22] where a delay of up to 24 hours was reported. Those who die before investigations and admittance into the ward are normally not diagnosed with TSCI before death and this adds to the missed cases. In Estonia for example, it is reported that up to 53% of patients with TSCI die before getting to hospital [23]. A prospective study that includes admission and follow-up of newly injured persons would provide more conclusive epidemiological data for Kilimanjaro. Establishment of regional and national electronic database for TSCI would benefit future retrospective studies and evaluation of strategies to prevent occurrence, morbidity and mortality due to TSCI.

Falls (especially from height) is the major cause of TSCI in this region, which is also as reported in some parts of India, Estonia, Pakistan, Turkey, Oceania and southern Asia [6,11,24,25]. Climbing trees in Kilimanjaro region is commonly done by male members of the family to obtain firewood, fruit and branches to feed cattle kept indoors. The female family members are responsible for carrying heavy loads of bananas, animal feed, firewood and other farm products on their heads to the home or market. It follows that TSCI following fall with heavy load on the head is more common among female population of this region than to their male counterparts. Based on these facts, prevention of falls in this region should consider the fact that the trauma-precipitating activities are motivated by both culture and economy [26]. It is important that the inhabitants of north-east Tanzania are well informed that falling either from trees or with heavy loads on the head adds to their risk of TSCI but also enabled to run safer socioeconomic and cultural activities.

The most affected age group (31–45 years) in this study is similar to most other studies [19,24,27]. Reason for relatively younger males involvement can partly be described by the etiology in this study where fall from height is the leading cause. Culturally, teenagers and young male in this region climb trees more frequently as they are considered stronger and safer while on the trees than the older men. This increases their risk of fall from height and TSCI consequently. Suffering TSCI at such a young age means many years lost to disability and reduced possibility to follow one’s career to contribute to the family and national economy. Students with TSCI fail to continue with school due to the restrictive environment and newly married and young parents fail to meet the needs for their families. Consequently, poverty strikes the family, children’s education is deterred and long term poor quality of life is inevitable hence a poverty-disability viscous cycle [28]. In this age group some are injured in the process of establishing a family or soon after marriage, which affects their sexual life leading to massive psychological disturbance [29].

Like in many low and middle income countries [4,30] pressure sores were the most prevalent complications in this study. Lack of pressure relief knowledge, skills, manpower and necessary tools have been identified as factors to the increase of pressure sore incidents [4,14]. The consequences of pressure ulcers include increased LOS, re-hospitalization and increased rehabilitation expenses. Pressure ulcers are also a common cause of death to persons with spinal cord injury in many lower income countries [4,14,31]. The magnitude of pressure ulcers, respiratory complications and urinary tract infections partly explain the high in-hospital mortality of 24.4% of all TSCI admissions [32]. Mortality could also have been instigated by associated injuries as LOS was found to be significantly shorter for persons who had associated injuries involving mostly head and chest. In addition the majority of the injuries were incurred at the cervical spine level which have detrimental immediate effect on the respiratory and autonomic nervous system which may have led to death in the first few days [33]. Emergency and intensive care services in Tanzania (particularly KCMC) are still insufficient for management of complex conditions such as high tetraplegia [20]. However, the scope of this study does not allow for determination of the exact cause of death. Generally, in order to reduce in-hospital mortality, comprehensive rehabilitation should complement a well-established on site casualties evacuation and transportation system as well as emergency and intensive care services. Continuous training of the health professionals and care providers regarding pressure ulcers prevention and provision of tools for skin, urinary and respiratory care would reduce complication and death incidents.

Of the patients who survived to discharge, 24.4% were wheelchair dependent. Appropriate wheelchairs, which are a prerequisite for mobility and function, are hard to obtain and afford in most lower income countries [34], even worse in the rural settings. In Tanzania particularly the setting for this study, more days are spent in the hospital by persons with TSCI awaiting a donated or subsidized wheelchair, as they cannot afford one by themselves. There was a significantly longer mean LOS for the wheelchair-dependent group (p ≤ 0.001) when compared to those who did not need one. Increased hospital stay and bed rest consequently adds to the risk of complications such as pressure ulcers, urinary tract infections and hypostatic pneumonia especially in the older patients and eventually death [14,32,33]. Appropriate wheelchair obtained earlier will enable the individual to be mobile, functional, productive and to interact with the social and physical environment from earlier stage. Sustainable wheelchair services are needed in the major hospitals of Tanzania, bearing in mind that almost a quarter of all those who suffer TSCI will require one whilst the majority cannot afford.

This study captured basic information regarding TSCI in north-eastern rural Tanzania. . This report is also a very important document which addresses falls as a major cause of TSCI in a rural setting for a more informed and focused prevention strategies.

### Strength and weaknesses

The strength of this study is that it is conducted in a one and only consultant hospital in the North-east Tanzania, serving a mostly rural population of about 11 million people. It is the only hospital in the country with a SCI unit and interdisciplinary rehabilitation team which started to operate officially in 2014. The study covers records of five consecutive years with a reasonably good sample size despite the missing records.

The major weakness of this report was the lack of comprehensive data collection on all cases of TSCI admitted in the specified period. This could make numeric calculations inconclusive and unrepresentative of the studied population. Furthermore, there was either absence of information or inconsistency in the use of internationally recommended patient progress assessment tools for SCI in most of the records. This inconsistency made it difficult to report clinical outcomes in accord with other published studies.

### Conclusion

The major cause of traumatic spinal cord injury in the North-east Tanzania is falls, particularly from height and mostly affects teenagers and middle-aged male. Prevention strategies in this region should target fall from height especially among teenage male and young adults. The major drawback of a retrospective study in this setting is missing cases and patient records leading to statistically inconclusive epidemiological results. Prevention of falls in Kilimanjaro region ought to target both risky culture and socioeconomic activities by informing and advising people on the risk of climbing trees and head lifting. Pressure ulcers, respiratory complications, in-hospital mortality and availability of wheelchairs should be addressed.

## Paper context

This study was conducted at Kilimanjaro Christian Medical Center (KCMC) which is a consultant hospital in the North-east Tanzania (bed capacity, 630). Kilimanjaro region (13,209 sq. km land; population 1,640,087) is a largely rural area. In 2014 KCMC opened a new spinal unit, (bed capacity, 25) for persons with traumatic SCI. However, no study has ever been published on magnitude and causes of traumatic spinal cord injury in this region.
